# A Synopsis of Contemporary Anesthesia Airway Management

**Published:** 2019-01-15

**Authors:** Christian Bohringer, James Duca, Hong Liu

**Affiliations:** Department of Anesthesiology and Pain Medicine, University of California Davis Health, Sacramento, California, USA

**Keywords:** Difficult intubation, Bougie, Video-laryngoscope, Fiberscope

## Abstract

New airway equipment has recently become available that has reduced morbidity and mortality. However, airway disasters still occur. This article discusses the prudent escalation of the use of advanced airway equipment to prevent these disasters. We illustrate when and how to use a gum elastic bougie and a video-laryngoscope (VL). We also strongly recommend the combined use of the VL together with a flexible intubation scope (FIS) for both asleep and awake intubation when dealing with a genuinely difficult airway. Blind intubations should no longer be performed today. When an airway has been recognized as difficult it is the safest to aim for an awake or at least a spontaneously breathing intubation if circumstances do not allow for an awake intubation. Emergency cricothyroidotomy needs to be prepared for so that it can be executed rapidly in case the attempted awake intubation leads to complete airway obstruction.

## Introduction

The availability of new airway equipment has been expanding at a steady pace in recent years not only in the operating room but also in the pre-hospital environment and in the critical care unit [1,2]. Many new methods of viewing the opening of the larynx have been developed and the incidence of catastrophic airway events has been reduced [3,4]. With the advent of these new devices some new problems like palate injuries have however become apparent. This article will focus on the prudent escalation of the use of advanced airway devices in the setting of a difficult intubation and how to avoid the dreaded “cannot intubate and cannot oxygenate” (CICO) scenario.

## Identification of a Difficult Airway

The first step in the prevention of airway disasters is the prediction of difficulty of intubation during standard direct laryngoscopy (DL). This has been traditionally accomplished by looking at mouth opening, neck motion, thyromental distance, overbite and mobility of submandibular soft tissue structures. The prediction of the difficult airway is unfortunately unreliable even when experienced clinicians are involved in the assessment and all of the above factors are considered in combination. A recent article on bedside predictors of difficult intubation found that current bedside tests have limited and inconsistent capacity to discriminate between patients with difficult and easy airways and that reliable bedside criteria to predict difficult intubation remain elusive [5]. Only about half of the difficult intubations were anticipated following a standard airway exam [6,7]. This means that an unexpected poor view of the vocal cords during standard DL will continue to occur. It is therefore imperative to focus strongly on the ready availability of advanced intubation and ventilation equipment in the operating room. When trying to prevent airway disasters the immediate availability of adequate airway equipment in the operating room will be more effective than conducting a thorough pre-operative airway exam. At the present time there is significant interest in the use of ultrasound to aid in the prediction of a difficult airway [8–10]. Other ways to assess the airway pre-operatively include X-ray, computed tomography with 3-D facial reconstruction and 3-D ultrasound reconstruction. It is, however, unlikely that the reliability of predicting a difficult intubation based on a pre-operative airway exam will improve much in the future. Currently, the best predictor for a difficult intubation is a previous history of difficult intubation in the hands of an experienced practitioner. Difficult airway registries have been developed to communicate difficult airway information to subsequent practitioners that will be managing the patient’s airway. These registries are especially important because the standard airway exam is so unreliable for predicting a difficult intubation.

## Preparation for Intubation

**Figure 1:**
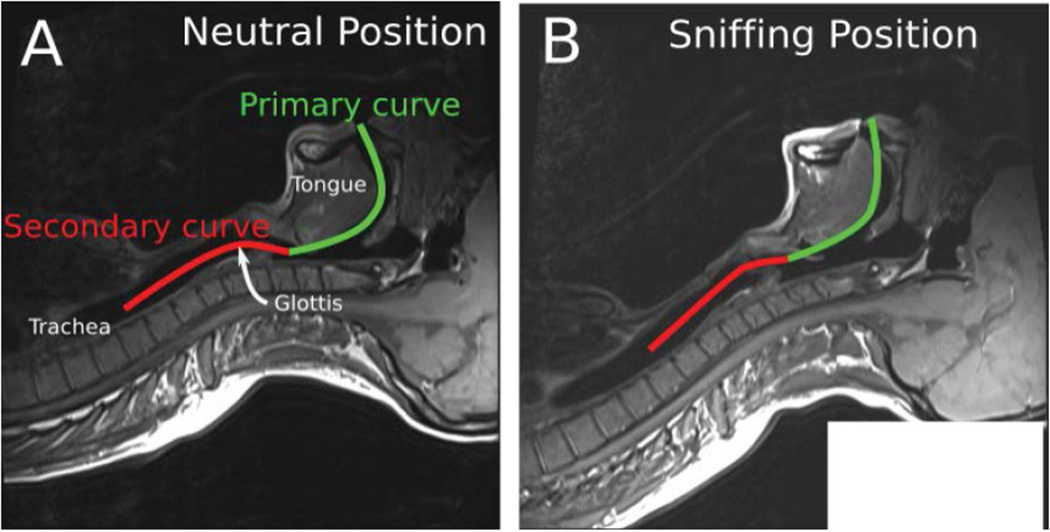
Alignment of the oral, laryngeal and pharyngeal axes with the intubator’s visual axis in the “Sniffing Position” as seen on an MRI scan [47]. Part of the figure was adopted from Greenland KB, et al. BJA. 2010;105(5):683-90. published by Elsevier with permission.

A review of the conditions for the initial laryngoscopy should be performed. The patient should be in the “sniffing” position with the neck flexed on the trunk and the head extended on the neck. This will align the oral, pharyngeal and laryngeal axes as much as possible (Figure 1). If the patient is obese, they should lie on a ramp to optimize the view at laryngoscopy.

It is undesirable to have a partially asleep and partially paralyzed patient because intubation will be difficult and mask ventilation may be difficult as well because the patient may subconsciously resist attempts at oxygenation via facemask ventilation. There is also the added risk of awareness for the intubation process. Intubation is one of the most common events remembered during cases of inadvertent intraoperative awareness. It is important to understand that the patient needs to be fully anesthetized and paralyzed before the first DL is performed [4]. Significant tachycardia and hypertension during attempted intubation are usually signs of inadequate depth of anesthesia and more intravenous induction agent should be administered. Eye evaluation should show the patient’s pupils are small and the eyes are non-divergent. Absence of complete neuromuscular blockade during intubation has also been associated with increased tachycardia and hypertension. Nandi and colleagues noted that intubation conditions were worse and there was more tachycardia and hypertension during intubation when intubation proceeded according to clinical impression of adequate relaxation rather than by verifying the presence of neuromuscular blockade with a nerve stimulator [11]. This increased heart rate and blood pressure may have resulted from the increased force required during laryngoscopy to expose the larynx when neuromuscular blockade had not been fully established yet. An adequate neuromuscular blockade during intubation was of utmost importance to avoid undue stimulation of the sympathoadrenergic system and they recommend the routine use of neuromuscular monitoring prior to intubation. An electroencephalogram (EEG) based neuromonitoring, like SedLine and bispectral index (BIS) monitors, can also be used to ensure adequate depth of anesthesia. In Nandi’s study the BIS index of both groups was comparable, but the adequately paralyzed group had less tachycardia and hypertension.

## First Direct Laryngoscopy

The first attempt at intubation should always be the best and everything should be optimized prior to having a look with the laryngoscope. All too often intubation is attempted before intravenous induction agents have fully crossed the blood brain barrier and neuromuscular blockade has taken complete effect. The result is a patient that closes the vocal cords during the intubation attempt. This impatience leads to multiple attempts at intubation and precipitates salivation and bleeding. When the airway is genuinely anterior this can then make the difference between success and failure when intubation has to be performed with VL and/or flexible intubation scope (FIS) techniques following the initial attempt using conventional DL.

In the past, it was recommended to limit the attempts at direct laryngoscopy to three times before progressing to an alternative intubating technique. In the hands of an experienced practitioner nowadays however a single attempt at direct laryngoscopy with optimal external laryngeal manipulation should be enough to ascertain that a VL and/or a FIS will be needed. Repeated attempts at standard DL need to be especially avoided in patients on anticoagulants, patients that will be given anticoagulants intra-operatively, patients on steroids and patients with significant ischemic heart disease. It can be argued that in this day and age the first attempt at direct laryngoscopy should not only be the best attempt but should be also the only attempt. If preparation for intubation has been adequate it is pointless to repeat the same intubation technique and expect a better outcome on the second attempt. Repeated attempts using direct laryngoscopy will produce saliva and bleeding and make eventual intubation with a FIS more difficult. They will also lead to laryngeal edema that will not only make intubation more difficult but may precipitate the CICO scenario. If laryngeal edema has occurred the treatment is intravenous dexamethasone and nebulized racemic epinephrine. Further attempts at conventional DL under these circumstances must be avoided at all cost. Laryngeal edema is usually diagnosed by the presence of inspiratory stridor and this problem needs to be monitored for in the post anesthesia care unit in any patient that had multiple DL attempts in the operating room. If stridor occurred in the recovery room and nebulized racemic epinephrine was administered, the patient needs to be admitted to the hospital because the stridor is likely to return when the effect of epinephrine wears off. It cannot be emphasized enough that this problem is largely preventable by employing a VL and/or a FIS sooner rather than later. Our mission as anesthesia care providers is not only to intubate successfully but also to know when intubation is truly necessary and how to intubate without causing any patient morbidity.

When difficult intubation is anticipated many authors recommend trans-nasal humidified rapid-insufflation ventilatory exchange (THRIVE) [12]. THRIVE administers humidified 100% oxygen at a flow rate of 70 L/min via nasal cannula. This set up is beneficial because the nasal cannula is out of the way and does not interfere with oral endotracheal intubation. Using THRIVE the median apnea time could be extended to 14 minutes. The main mechanism of this impressive extension of apnea time by THRIVE is probably apneic oxygenation in the presence of a patent airway but other mechanisms may be involved as well. When difficulty is encountered THRIVE will buy a lot of extra time before hypoxemia occurs. If THRIVE is not available in the operating room a conventional nasal cannula at an oxygen flow rate of 15 L/min can be used keep the patient oxygenated during apnea in case of a prolonged and difficult intubation.

## Management after an Unexpectedly Poor View of the Vocal Cords with Standard DL

The first step after identifying a poor view of the vocal cords at laryngoscopy should always be to ventilate the patient and call for help [4]. The emphasis needs to be on maintaining a patent airway and continuous oxygenation of the patient. Intubation is not really necessary for many operations and a supra-glottic airway (SGA) device such as a laryngeal mask airway (LMA) will be an adequate airway for the conduct of the entire procedure. The anesthesia care provider needs a high degree of flexibility of mind and needs to be able to reassess if a deviation from the original anesthesia plan would be in the patient’s best interest. The surgeon should be included in the airway management plan as well because changing the operating position from prone to lithotomy for example would make intubation less important. The surgeon also needs to be included in this discussion because she/he may well be called upon to perform a cricothyroidotomy if things end up not going well.

The presence of a second pair of skilled hands is absolutely invaluable during a difficult intubation scenario. The anesthesia care provider’s ego needs to be put aside and help needs to be requested as soon as the difficult airway has been recognized. As professionals we owe our patients a duty of care to work cooperatively as a group to ensure the best outcome possible for them. When coming into another operating room to assist another care provider with an unpredicted difficult airway it is very important to be helpful to the process. The helper needs to ascertain what the airway situation is like and what can be done to improve the situation. The helper should make every attempt to find out what the current plan is and then help the primary anesthesia provider carry out that plan. Comments like “why are you doing something stupid like this” and “I would have never managed this case this way” are not really helpful in this situation. A helper is also of great benefit to the process because the primary anesthesia care provider may be overly stressed by the situation and high circulating catecholamine levels may make him/her regress and lead to the same technique being tried over and over again. This will lead to laryngeal edema and the CICO scenario. If this should occur the helper needs to step in and stop this cycle to prevent a bad patient outcome. Appropriate helpers would be another anesthesiologist, nurse anesthetist or an anesthesia technician. It is highly desirable that the helper is intricately familiar with anesthesia airway equipment and intubation and oxygenation procedures.

If there is any concern about the effectiveness of mask ventilation or successful ventilation via an LMA, it is the safest option to wake the patient up and plan for an awake flexible scope intubation (FSI) [4]. Insertion of an oral, nasal or laryngeal mask airway may be required to keep the patient oxygenated until the neuromuscular blockade has been reversed. The recent admission of sugammadex, a rapidly acting specific reversal drug for rocuronium, to the US market has made this process much easier than before. Sugammadex has been a giant step forward in reversing neuromuscular blockade rapidly [13]. Even though it does not appear to block the anaphylaxis in cutaneous models there are many case reports that describe a dramatic attenuation of the anaphylactic reaction that is occasionally seen with the administration of rocuronium. If anaphylaxis to rocuronium is suspected rapid high dose sugammadex reversal is currently recommended [14–17]. Succinylcholine is rarely used now since this rapid reversal option for rocuronium neuromuscular blockade has become available.

If mask ventilation is considered to be adequate serious consideration should be given to changing the original anesthesia plan and proceeding with an LMA anesthetic. Proceeding with intubation attempts in the setting of an anterior airway and poor view at laryngoscopy is associated with a higher incidence of airway trauma and vocal cord damage than in a patient with conventional airway anatomy. Endotracheal intubation should therefore only be performed in this situation if it is really necessary. Many cases of post-intubation airway damage in patients with an anterior airway could have been avoided if the case would have been managed with SGA. When managing difficult intubation cases with SGA, however, it is important to maintain adequate depth of anesthesia throughout the surgery to prevent the occurrence of laryngospasm.

Many operations and clinical scenarios have an absolute requirement for endotracheal intubation. When endotracheal intubation is truly necessary advanced airway techniques will become necessary. It cannot be overemphasized that the first priority during airway management is to always keep the patient oxygenated to prevent hypoxic encephalopathy rather than to successfully intubate the trachea. An LMA should be used whenever there is any difficulty with adequately oxygenating the patient during facemask ventilation. The LMA is now accepted as the first line aid to keep the patient adequately oxygenated.

## When to use a Gum Elastic Bougie

The first line aid to difficult intubation on a worldwide basis is the intubation guide commonly referred to as a gum elastic bougie. This name does not really describe this device well because it is neither made of rubber nor is it used to dilate any structures. It would be better to refer to it as an intubation guide but at the present time the name bougie is firmly entrenched in common everyday usage. The bougie should be used when only the arytenoids are visible or external laryngeal manipulation is required to improve the view during DL. It should not be used when the arytenoids are not visible and a VL should be employed then to ensure correct placement of the ETT during the first attempt. Blind intubations should no longer be attempted when a VL or a FIS is available. The bougie is very helpful to line up the axis of the endotracheal tube (ETT) with the axis of the larynx. In this way it functions very similarly to a wire in an arterial line kit that lines up the axis of the arterial line cannula with the axis of the artery. On occasions the bougie will pass through the vocal cords but then fails to advance. This is due to the tip of the bougie impinging on the anterior wall of the trachea. Rotating the bougie 180 degrees around its own axis will make the “Hockey Stick” portion of the bougie point posterior and allow the bougie to pass further into the trachea. This maneuver is much easier to perform when the bougie is placed on its own rather than when the ETT is already preloaded over it. In a difficult intubation scenario, the bougie should therefore be placed into the trachea by itself before sliding the ETT over it.

The ETT should always be lubricated whenever a difficult intubation is anticipated to allow for an easy advancement of the ETT over the bougie or the FIS into the trachea. It is essential that the laryngoscope blade remains in the pharynx with an appropriate amount of lifting force applied to the vallecula when the ETT is advanced over the bougie because this causes the line of the oral, laryngeal and pharyngeal axis to be as straight as possible. A standard ETT made of polyvinylchloride is a straight rigid structure that does not like to negotiate corners. It is therefore important that during ETT insertion all the airway axes are aligned as much as possible to ensure that this process takes place without causing damage to the patient’s airway. If there is a holdup when attempting to pass the ETT over the bougie, this is probably due to the ETT impinging on the right arytenoid. In this case the ETT should be withdrawn about one centimeter and then turned 90 degrees to the left before advancing it again. This allows the bevel of the ETT to clear the right arytenoid because it will be pressed against the bougie rather than against airway structures. The tip of the ETT usually impinges against the right arytenoid because the bevel and therefore the tip of the ETT are located on the right side of the ETT. Warming the ETT in sterile warm water or a heating cabinet will make it more pliable and allow it to follow a guide like a bougie or a bronchoscope more easily. Spirally reinforced “armored” ETTs and ETTs with tips made of silicon (like the intubating LMA ETT) have less endogenous rigidity and therefore will follow intubation guides more easily than conventional ETTs made of polyvinyl chloride. The bougie needs to be used with caution and it should never be used with force because inadvertent tracheal perforation has been described with its use [18,19]. The bougie can also be used as an aid to exchange an ETT when intubation has been difficult [20]. The bougie is the preferred intubation aid whenever external laryngeal pressure needs to be applied to the neck to improve the view at laryngoscopy. The bougie can be inserted while the pressure is still on the neck because it has a smaller diameter than the ETT. This allows it to pass the location at which the neck pressure is applied. After the bougie has been inserted however the neck pressure needs to be released so that the ETT can successfully slide over it.

If a standard stylet is used when external optimal laryngeal manipulation is applied to improve the view at laryngoscopy the tip of ETT will pass through the cords but will not pass the point at which neck pressure is applied. At this point, the force of the external laryngeal pressure narrows the diameter of the trachea because unlike the cricoid cartilage the trachea does not have a continuous cartilaginous ring holding it open. The external laryngeal pressure will therefore impede the passage of the ETT. When the external neck pressure is released to enable the passage of the ETT the anterior larynx will recoil back to its anterior position and the tip of the ETT frequently flops out of the larynx and the ETT will be inadvertently advanced into the esophagus. This often comes as a surprise to junior anesthesia care providers because they saw the tip of the ETT pass the vocal cords. The result will be an esophageal intubation that will only be identified after ventilation attempts have inflated the stomach with oxygen and the absence of end tidal CO_2_ on the capnograph has been noted. It is therefore highly recommended to use a bougie first whenever external laryngeal pressure has to be applied to improve the view of the vocal cords at DL. Esophageal intubation should be avoided because the resulting stomach inflation can lead to aspiration of residual stomach contents. If the stomach inflation is severe the left hemi-diaphragm will be elevated and the base of the left lung will collapse. This often leads to difficulty with oxygenating a patient when ventilating with the facemask after an unintentional esophageal intubation. This phenomenon is especially severe in neonates and small children and suctioning the stomach is of great priority in these patients. Stomach suction may need to be performed prior to correct endotracheal tube placement so that the patient can be adequately oxygenated via facemask ventilation. Residual gas in the stomach is also a significant cause of medication resistant nausea and belching in the recovery room. If stomach distension is severe this can cause a severe vagal response and may be associated with a bradycardic cardiac arrest. There has also been a case of ruptured stomach reported with inadvertent esophageal intubation [21]. Particular attention should therefore be applied to try to remove as much gas as possible from the stomach after an unintentional esophageal intubation has taken place. It needs to be emphasized that unintentional esophageal intubation is not a benign and harmless error even if it is recognized immediately and it should be avoided as much as possible. In cases where there is some doubt about ETT location it may be prudent to administer a single breath and wait for the capnograph confirmation rather than to pump several large tidal volume breaths into the patient’s stomach. If an ultrasound machine is in the room ultrasound can be used to confirm correct endotracheal intubation. This can prevent gastric insufflation in case of inadvertent esophageal intubation. The practice of using blind intubation attempts should therefore be discouraged. Blind intubation should only be attempted if advanced airway equipment is not available [4].

Better equipment is now readily available in most operating rooms to image the larynx during intubation and anesthesia care providers should be familiar with this equipment. Visualization of the larynx during intubation now really has become a standard of care and in capable hands the use of this equipment speeds up intubation and prevents airway trauma. When anesthesia care providers are facile with the use of a VL and a FIS and they use these in combination the time to intubate patients with a difficult airway has now been reduced significantly. It usually takes more time to get the equipment into the operating room than to perform the intubation. Many hospitals have been very proactive in providing advanced airway equipment because they know that in experienced hands the ready availability of this equipment saves a lot of operating room time. This time is at a premium and frequently waiting for a VL or a FIS to become available soon adds up to significant cost. There is also the added risk that anesthesia care providers will persist with their attempts at conventional DL while they wait for a VL or a FIS to arrive. This practice is very dangerous and anesthesia care providers need to learn to desist early at attempts at DL when faced with an unpredicted difficult airway.

## When to use a VL

When the arytenoids cannot be visualized at laryngoscopy even when optimal external laryngeal pressure is applied more advanced airway equipment such as a VL needs to be employed [22]. Repeated attempts at DL should no longer be performed in this situation. “Blind” intubation attempts with the bougie should also no longer be performed. With every subsequent laryngoscopy more salivation, bleeding and airway swelling will ensue. This will make subsequent visualization with advanced airway equipment more difficult and may even cause the larynx to swell up so much that mask ventilation will be no longer possible. Repeated attempts with DL may therefore precipitate the dreaded CICO scenario. There are a number of VLs on the market like GlideScope, C-MAC, KingVision, McGrath and Airtraq [23].

Given the recent advances in intubation equipment, visualization of at least the posterior part of the larynx while intubating should now be the standard of care because it allows for identification of any airway trauma from the intubation process. It also avoids esophageal intubation and insufflation of the upper gastrointestinal tract when the patient is ventilated to confirm the presence of CO_2_ on the capnograph. Visualization also helps to identify the cause for failure of the ETT to pass over a bougie or a FIS. When looking at the cords during intubation it will become obvious if the ETT is too big, is jamming against the right arytenoid or if the fiberscope has been pulled out of the larynx by the advancing ETT to form a blind loop.

Depending on the response time for difficult airway equipment to arrive in a particular institution it may be advantageous to insert an LMA and use the ventilator to ventilate the patient. This will prevent the hand and forearm muscles from becoming fatigued as a result of prolonged bag mask ventilation and maintain hand co-ordination. This hand co-ordination will be essential once advanced intubation equipment arrives in the operating room. Positive pressure ventilation via LMAs is now commonly employed in anesthesia but must be carried out with care to prevent stomach inflation [24,25]. The next step will be to attempt visualization of the larynx with a VL. Multiple devices have recently become available and they all afford a much better view of an anterior airway than a conventional DL.

VLs and their stylets have been designed to allow for intubation of patients with the neck in the neutral position. They have become very popular in the intubation of trauma patients that may have an underlying neck fracture. They are usually intubated with the neck in neutral position employing manual in line stabilization and a hard collar *in situ.* Given this design it is prudent to take the patient out of the sniffing position and place the head and neck in the neutral position to allow for a better alignment of the ETT with the larynx if the standard stylet for the VL is used. VLs are also useful when intubating patients without the aid of neuromuscular blocking agents because they do not require force to be applied to the vallecula to visualize the cords. They therefore are less likely to be associated with laryngeal spasm if the patient is in a light plane of anesthesia. VLs are also useful as a first line intubation method in patients with temporomandibular joint problems because less force needs to be applied to enable intubation (Table 1) [26,27].

With the advent of the VL some new problems have however emerged. Palate injuries were previously uncommon with the use of direct laryngoscopy (DL) but they now occur with the use of a video-laryngoscope (VL) [28–32]. These injuries are usually due to operator error. During intubation with a VL the intubator should watch the ETT entering the mouth and pharynx and only then look at the VL screen to place the ETT into the larynx [33]. Keeping the tip of the ETT in close proximity to the VL blade will reduce the likelihood of accidentally injuring the palate with the tip of the ETT. Insufficient training in the use of advanced intubation devices and lack of understanding of when and how to use these devices in isolation or in combination still leads to the occurrence of airway disasters.

**Figure 2:**
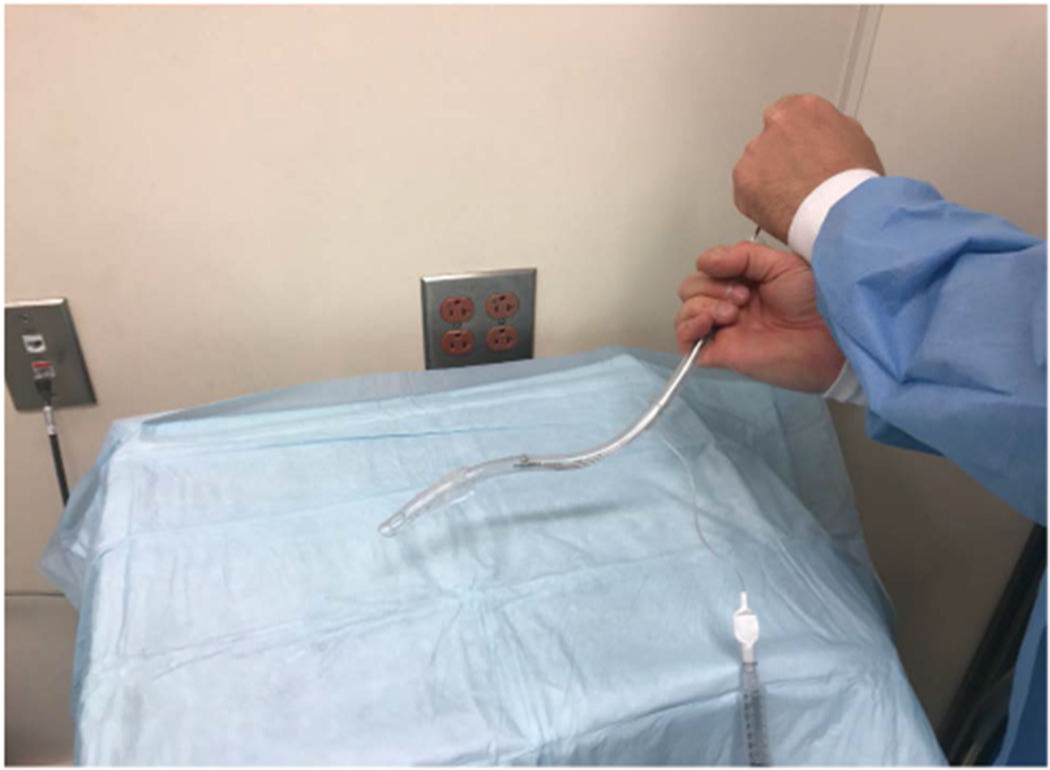
Retro-flexion of the tip of the endotracheal tube (ETT) when it is turned 180 degrees while holding the top of the stylet in place.

One of the inherent problems of VLs is that they allow the visualization of the cords by looking around the corner but they do not always allow for easy intubation because they do not line up the oral, laryngeal and pharyngeal axes as efficiently as a DL. This issue is particularly significant in patients with an anterior airway where the ETT first needs to ante-flex to enter the cords and then retro-flex to advance further into the trachea. This lack of aligning oral, laryngeal and pharyngeal axes during the use of a VL is probably the reason why a VL has not been found to be superior to a standard DL when used for routine intubation of patients without anterior airway [34,35]. During intubation with a VL the ETT will occasionally pass through the vocal cords but then stop advancing. This is especially likely when the patient has an anterior airway. The problem in this situation is that the tip of the ETT is lodged against the anterior wall of the trachea. In this case the ETT needs to retroflex after passing the vocal cords in order to line up with the axis of the trachea. In order to advance the ETT the stylet should be withdrawn 2-3 cm. An assistant should then hold the top of the stylet in place to stabilize it while the intubator is turning the ETT 180 degrees to the left in order to retroflex the tip of the ETT (Figure 2). This will align the axis of the ETT with that of the trachea and often allows for atraumatic advancement of the ETT into the trachea. ETTs should always be well lubricated whenever intubation difficulty is anticipated. Some practitioners reverse-load the ETT onto the VL stylet to achieve a similar effect.

## When to use a VL Together with a FIS

If the patient’s airway is extremely anterior the vocal cords can usually be well visualized with the VL, but it is often difficult to pass the ETT. If the tip of the ETT cannot be passed through the vocal cords with the standard stylet, a FIS should be used to steer the ETT into the larynx while looking at the vocal cords with the video-laryngoscope. The combination of a bougie with a VL is not recommended in this situation because the bougie cannot be steered into the airway like a FIS. The VL also provides less space in the mouth than a McIntosh blade and this makes manipulation of the bougie with McGill forceps difficult. While the VLs look around the corner to visualize the cords the bougie is fundamentally a straight device that does not want to negotiate corners. The FIS is far superior to the bougie in this situation because it is flexible and can be easily ante-flexed and retro-flexed. It needs to be emphasized that employing a FIS at this stage is essential to prevent intubation trauma. Thoughts like “but somebody will need to clean the FIS if we use it” need to be resisted at all cost. The anterior airway is a difficult management problem and the sooner the anesthesia care provider uses the “good equipment” the better off the patient will be. Repeated attempts at conventional DL techniques in this situation will make the patient salivate, bleed and cause laryngeal edema.

The outside diameter of the FIS should be at least three quarters of the internal diameter of the ETT. This will make it more likely that the ETT will follow the FIS into the trachea. If the FIS that is used is too small for the size of the ETT the inertia of ETT will pull the FIS out of the trachea to form a blind loop. The ETT will then not slide over the FIS into the trachea but will pull the FIS out of the trachea. The largest FIS that comfortably fits through the ETT should therefore be used as a guide for the ETT [36]. A pediatric FIS should therefore not be used to attempt to steer an adult size ETT into the trachea. A difficult airway management crash cart should include both a pediatric as well as an adult size FIS.

**Figure 3:**
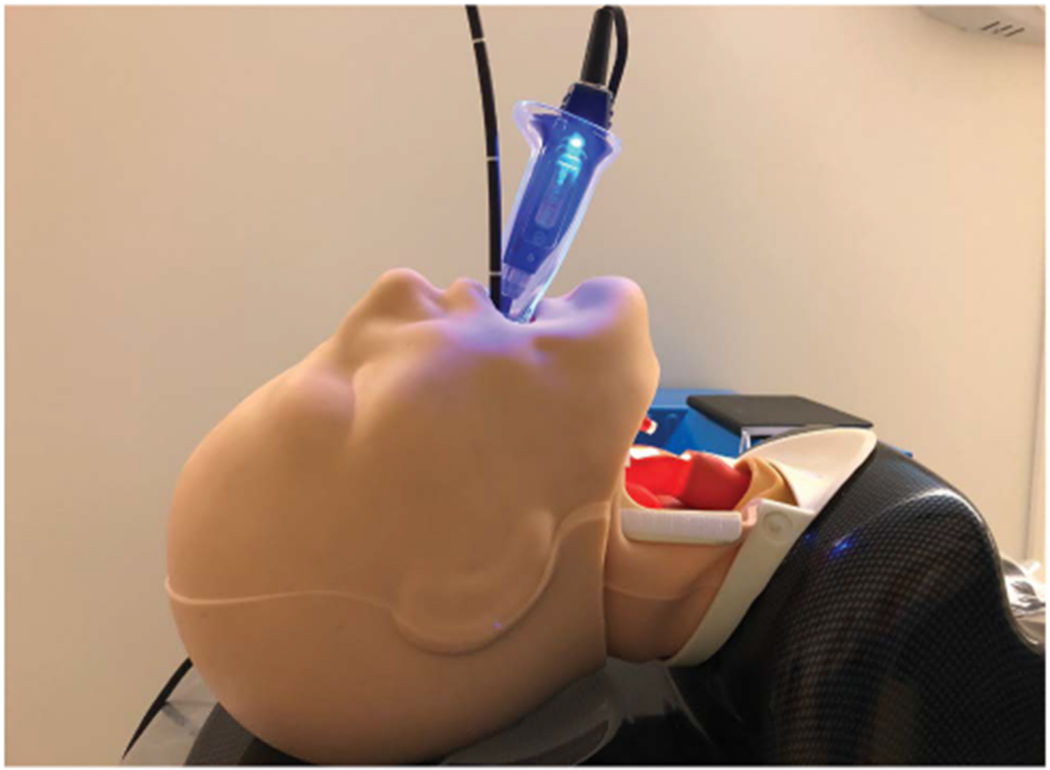
The vocal cords are visualized with the video-laryngoscope (VL) while the flexible intubation scope (FIS) is used to steer the endotracheal tube (ETT) into the trachea.

**Figure 4:**
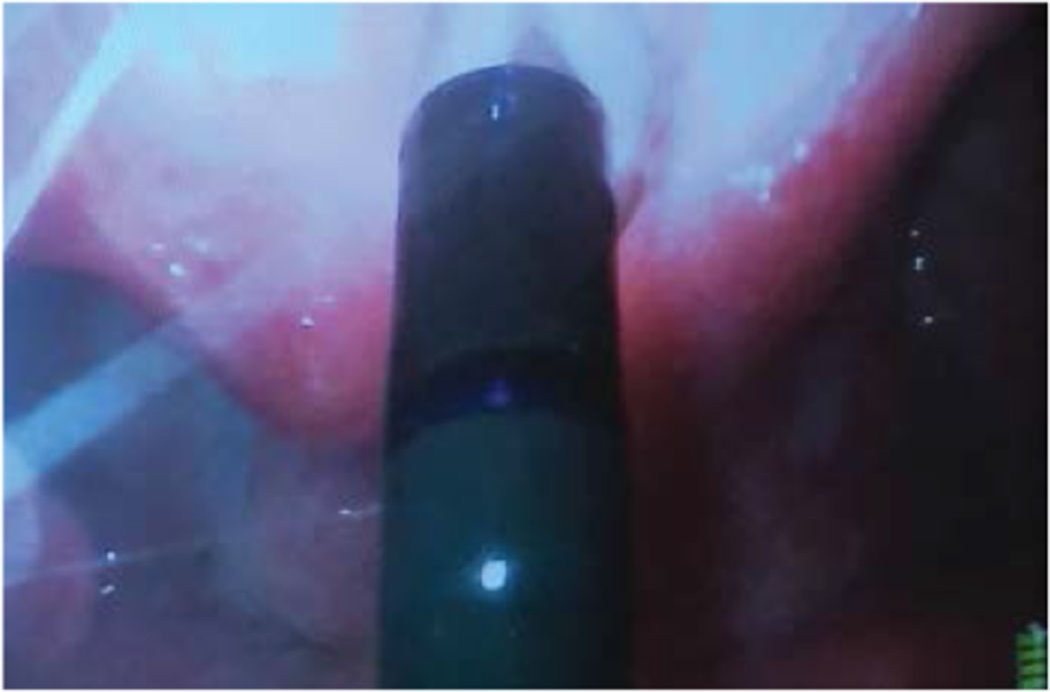
Video-laryngoscope (VL) view of the flexible intubation scope (FIS) having passed the vocal cords. The FIS is retroflexed by pushing the thumb up.

**Figure 5:**
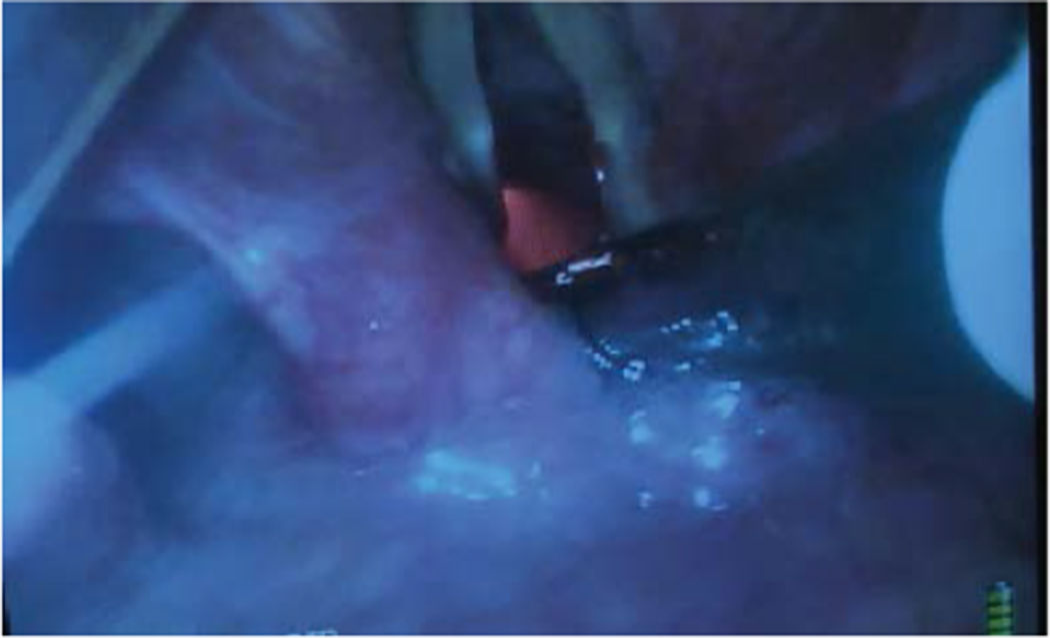
Video-laryngoscope (VL) view of the bevel of the endotracheal tube (ETT) impinging on the right arytenoid as it is attempted to pass it over the FIS into the trachea.

When the trachea is extremely tortuous and it has been impossible to slide a standard ETT over the FIS into the trachea, a spirally reinforced or “armored” ETT can be used. This type of ETT will follow the FIS more easily because it has less internal rigidity and therefore conforms more readily to unusual tortuous airway anatomy. The Parker flex tip endotracheal tube has also been shown to pass more easily over a fiberscope than a conventional ETT [37]. The ETT that is provided with the intubating LMA is a soft wire-reinforced silicon tube and its tip conforms readily to airway anatomy and is also excellent at sliding easily over a flexible bronchoscope (Figure 3, Figure 4 and Figure 5) [38].

**Figure 6:**
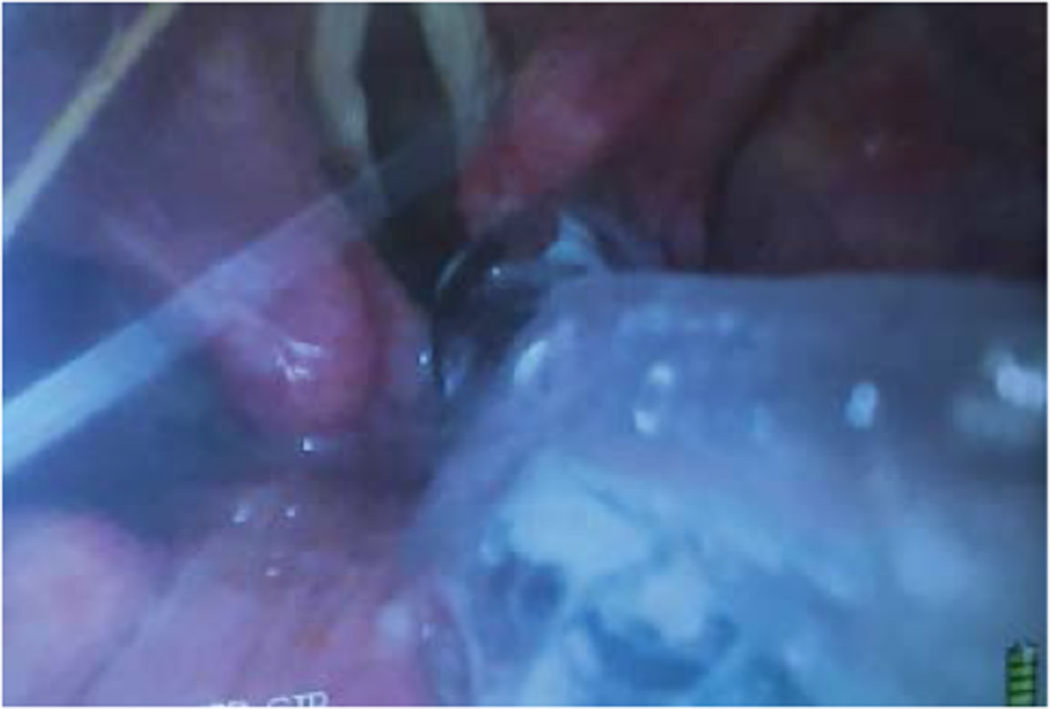
Video-laryngoscope (VL) view of the bevel of the endotracheal tube (ETT) impinging on the right arytenoid as it is attempted to pass it over the FIS into the trachea.

It is highly desirable to combine the use of the VL together with the FIS in this situation. Conventional FIS intubation is really a form of blind intubation. Even though the camera at the tip of the FIS confirms the tracheal position of the tip of the FIS there is no visualization of the vocal cords as the ETT is passing through them. When the airway is very anterior it is not uncommon to have a holdup of the ETT as it is attempted to be passed through the vocal cords. The potential for vocal cord trauma during this maneuver is very significant. Looking at the vocal cords with the VL as the ETT passes over the FIS will identify any damage that is occurring to the vocal cords. It also identifies the cause of any holdup that may be occurring. The VL view may also be used to identify maneuvers that may aid in getting the ETT to pass through the cords. Looking at the vocal cords during FIS intubation will make it obvious if the ETT is sufficiently aligned with the trachea and if anterior neck pressure should be applied to help guide the ETT into place. With this set up the intubator gets visual feedback about what effect manipulating the top of the ETT has at its distal tip. A common cause for failure of the ETT to slide over the FIS into the trachea is that the bevel of the ETT is impinging on the right arytenoid. In this situation pulling the ETT back 1 cm and turning the ETT 90 degrees to the left prior to advancing it again will allow the ETT to pass into the larynx. This 90-degree turning maneuver to the left allows the bevel of the ETT to clear the right arytenoid as it is pushed centrally between the cords against the FIS (Figure 6).

**Figure 7:**
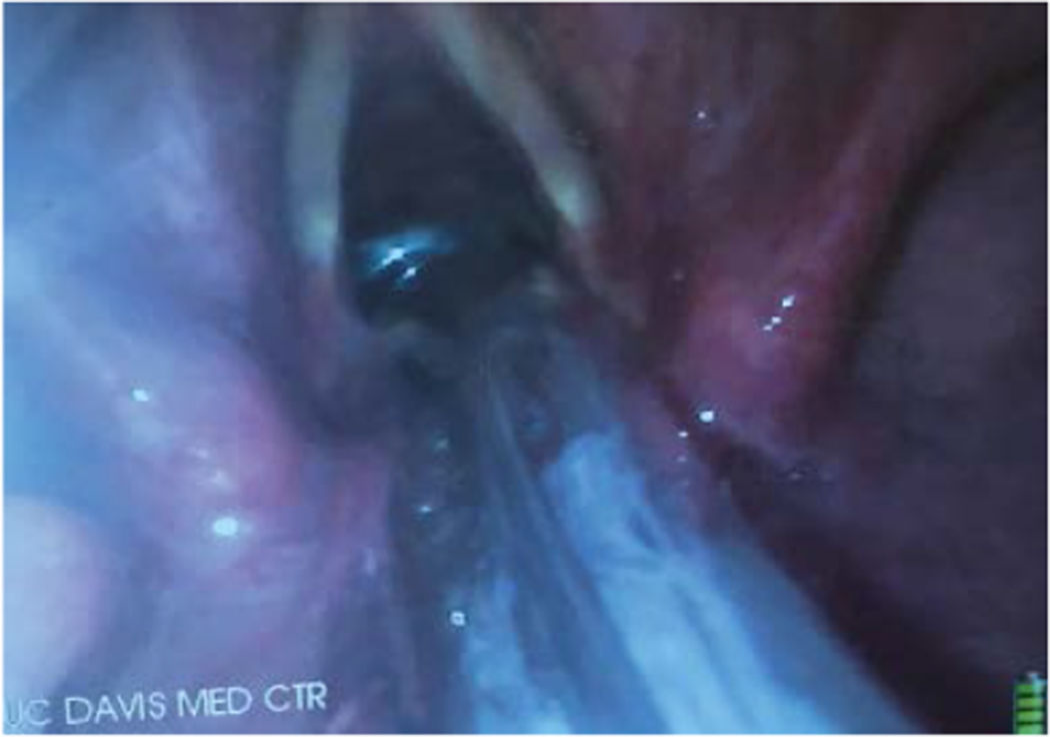
Endotracheal tube (ETT) cuff inflated just below vocal cords as visualized by the video-laryngoscope (VL).

Pulling the ETT back one centimeter and turning it to the left will allow it to successfully clear the arytenoid and pass into the trachea. The use of the VL allowed the anesthesiologist to diagnose the cause of the holdup and prevented potential laryngeal damage. Turning the ETT 90 degrees to the left is a well known technique to prevent impingement of the ETT on the laryngeal inlet [39]. Another cause for holdup is the blind loop syndrome when the ETT pulls the FIS out of the trachea to form a blind loop. This usually occurs when the diameter of the FIS is too small for the diameter of the ETT. Again, this is readily diagnosed by looking at the cords with the VL. Occasionally, the ETT will not pass because the diameter of the ETT is too large for the patient’s larynx. All of these causes of failure to successfully slide the ETT over the FIS into the trachea can be readily identified by the view obtained with the VL. The combined use of the VL together with the FIS is therefore preferable over the use of the FIS on its own. It greatly reduces the potential for airway trauma. It also allows the visualization of the ETT cuff just below the vocal cords and avoids inadvertent endo-bronchial intubation. This is a great advantage over standard DL when the cords cannot be visualized because following a difficult intubation the ETT is often inadvertently advanced into the right main bronchus. After a difficult struggle to insert the ETT into the trachea the anesthesia care provider wants to make sure the ETT does not accidentally get dislodged out of the trachea. In an attempt to prevent this the ETT is often advanced too far resulting in an endobronchial intubation. The poor view of the larynx with DL did not allow the cuff to be visualized just distal to the vocal cords. VL is helpful here because the cuff of the ETT can be seen just distal to the vocal cords (Figure 7).

**Figure 8:**
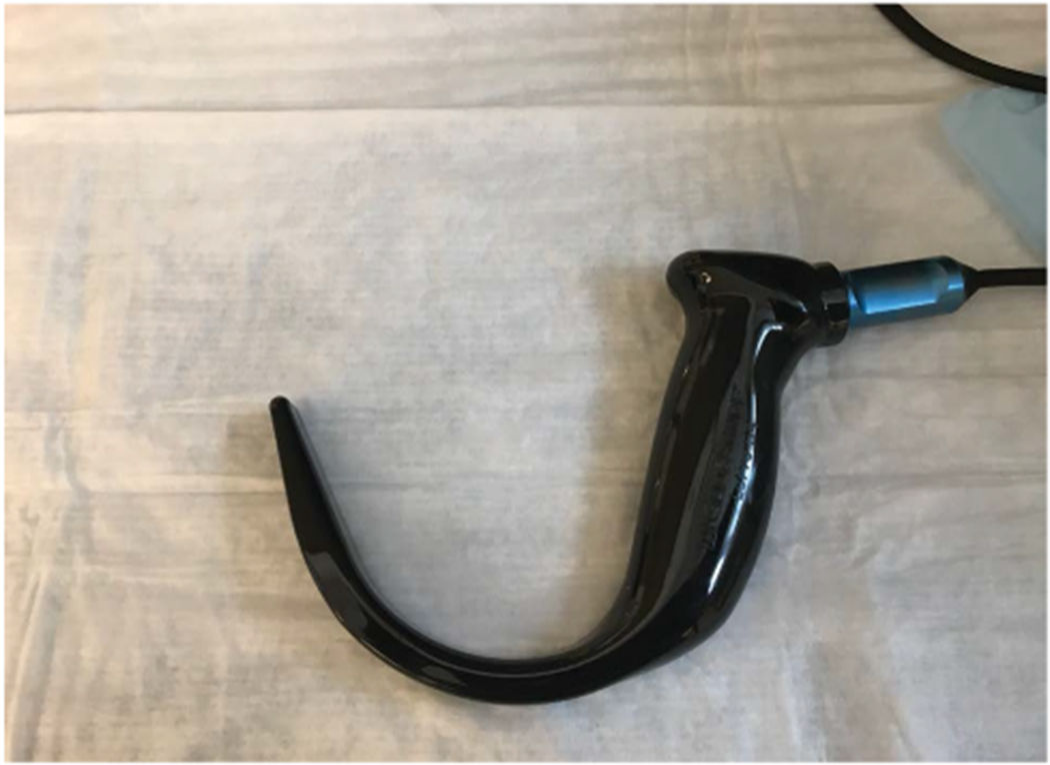
Low profile video-laryngoscope (VL) blade for use with limited mouth opening.

This combined technique of using the VL in combination with the FIS is also of great use when intubation has to be performed awake. Visualizing the vocal cords with the VL in an awake patient much better tolerated than with a conventional DL. With the VL there is no need to apply force to the vallecula to obtain a view of the vocal cords. So, it is much less likely to make the patient gag or precipitate laryngospasm. This also makes the VL an opportune intubation aid when endotracheal intubation has to be performed in the absence of neuromuscular blocking agents. Low profile blades for VLs are now available. They have been designed for use in patients with limited mouth opening. The low-profile blades allow a VL blade to be inserted into the patient’s mouth as long as mouth opening is wide enough for the ETT to pass (Figure 8). These airway management techniques can also be used in children and low profile VL blades can be used even in small children. The availability of low profile VL blades now relegates the conventional FIS intubation to cases where there is absolutely no mouth opening. The most common scenario of this is when the mandible has been wired to the maxilla.

**Figure 9:**
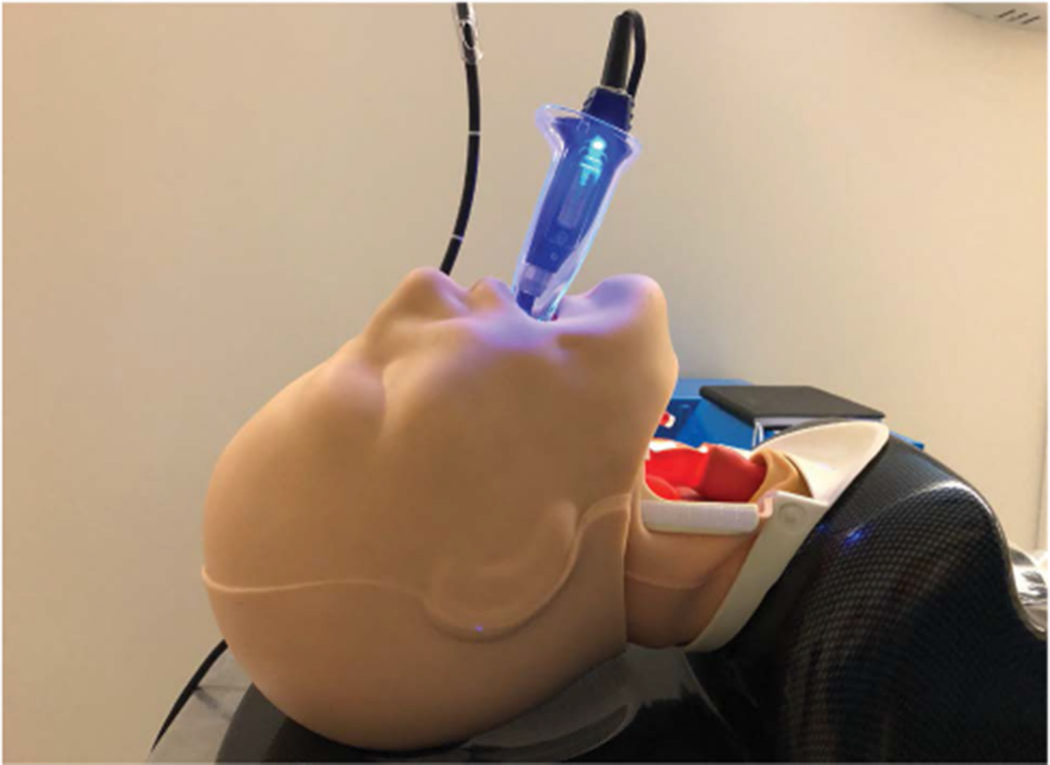
The vocal cords are visualized with the video-laryngoscope (VL) while the flexible intubation scope (FIS) is inserted through the nose to steer a nasal endotracheal tube (ETT) into the trachea.

The combined use of the VL with the FIS is also useful for nasal intubations. When the neck has not been cleared in patients with mandibular fracture this technique allows the passage of the nasal ETT with minimal neck movement. In patients with an anterior airway this technique allows for an easier placement of the nasal ETT because the fiberscope can be ante-flexed and easily steered into the trachea. If there is blood in the airway in trauma patients the view from the VL is usually much less impaired by it than the view from the FIS (Figure 9). When using the VL together with the FIS it is not always necessary to connect the optical portion of the FIS. Since the visualization of the vocal cords is achieved with the VL the FIS can be used as a steerable bougie or intubation guide for the ETT without using the camera at the tip of the FIS. In high risk awake intubations, it is, however, advisable to also connect the FIS to a camera in case the VL view is limited by patient discomfort. Patient discomfort is usually due to inadequate preparation with local anesthetic.

Intubating an awake patient with this combined technique has several advantages. The blade of the VL acts like an oral airway and lifts the tongue forward and prevents airway obstruction. This is of great benefit in sleep apnea patients. The blade also protects the FIS from the patient biting on it in case the topical anesthesia with local anesthetic has been suboptimal. There are case reports using this combined technique in the recent literature [40,41]. Dexmedetomidine has been recognized as an excellent adjunct to local anesthesia for awake intubation. It is a powerful anxiolytic, has minimal respiratory depressant effects and suppresses airway reflexes like gagging and laryngospasm. It also prevents tachycardia and hypertension during the intubation process. It has been found to be superior as a sedative to fentanyl, remifentanil and midazolam for awake FSI [42–44]. Whenever an awake FSI is planned there also needs to be a backup plan to be able to rapidly proceed to an invasive percutaneous airway in case the awake intubation attempt precipitates complete airway obstruction. This can be done with a needle cricothyrotomy which is preferred in prepubertal children, a Seldinger-style over the wire cricothyrotomy or an open cricothyrotomy utilizing a scalpel. A bougie can be useful to ensure the correct placement of the ETT during an open cricothyrotomy. The exact mode of management will depend on the type of equipment that is immediately available and the caregiver’s familiarity with a given technique. A formal cricothyrotomy with a cuffed ETT or tracheostomy tube is however preferred if there is a risk of aspiration.

An awake intubation attempt is safer than an attempt under general anesthesia and neuromuscular paralysis, but complete airway obstruction may still occur [45]. This is particularly likely if signs of airway obstruction are already present even prior to the intubation attempt. On occasion the mere application of topical local anesthetic to the airway may be sufficient to precipitate complete airway obstruction. Adequate local anesthesia of the airway however is an absolute prerequisite when performing an awake intubation to prevent laryngospasm, tachycardia and hypertension. This can be achieved with nebulized lidocaine, spray as you go or nerve block techniques. If airway obstruction is complete an expeditious surgical cricothyroidotomy will then become necessary. Awake intubation attempts in patients with stridor should only be performed in locations where a surgical cricothyroidotomy can be performed quickly and a capnograph for continuous CO_2_ monitoring is available. The best way to perform a cricothyroidotomy has been described in detail elsewhere [46]. It needs to be emphasized that while an awake intubation is often the safest management for the patient with a difficult airway, complete airway obstruction can still occur when attempting it. Every anesthesia care provider therefore needs to be technically competent to perform a cricothyroidotomy and insert an oxygenation device. With the judicious and rapid escalation in the use of advanced airway equipment described in this article the need for an emergent cricothyroidotomy should however be reduced in the future.

## Summary

**Figure 10:**
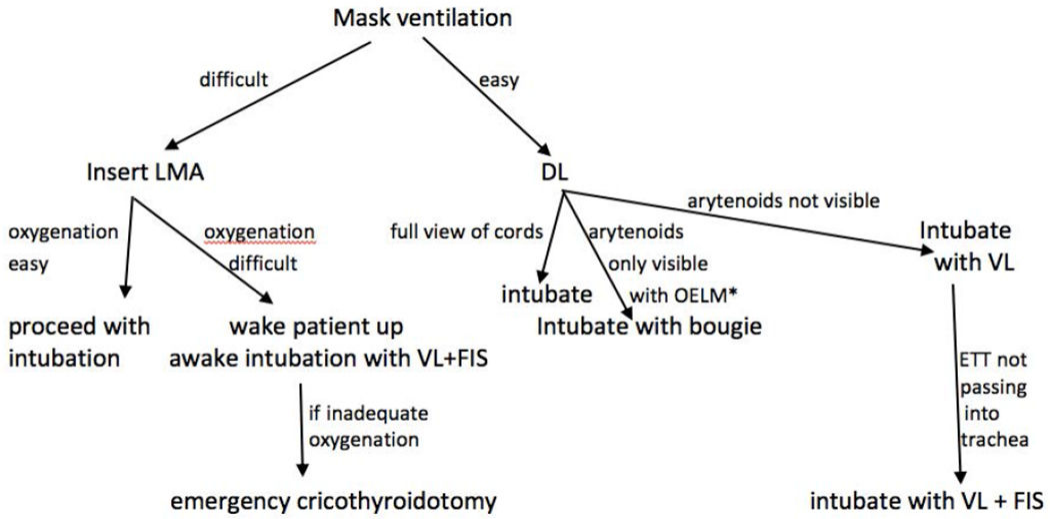
Airway management flowchart. OELM: Optimal external laryngeal manipulation; DL: Direct laryngoscopy; LMA: Laryngeal mask airway; VL: Video laryngoscopy; FIS: Flexible intubation scope.

New airway devices have recently become available and anesthesia care providers need to become facile with their use. Given the difficulty of adequately predicting a difficult airway pre-operatively, the ready availability of these devices in the operating room is very important. Having this equipment on standby in the operating room is of greater benefit than a thorough preoperative airway evaluation when the aim is to avoid airway disasters. Advanced airway devices should be used early and repeated attempts at direct laryngoscopy should no longer be employed. When contemporary airway equipment is available in the operating room, blind intubation should no longer be performed. Esophageal intubation is not harmless even when recognized quickly and should be avoided. Visualization of the vocal cords during intubation will not only eliminate esophageal intubations but also reduce trauma to the vocal cords that is likely to occur during the intubation of patients with an anterior airway. The combined use of a VL with a FIS is a useful technique when the ETT cannot be passed with the standard stylet for the VL. The combined technique can be used for both asleep as well as awake FSI. These airway techniques should be taught to residents and all other anesthesia staff in simulation centers to ensure that they can be performed expeditiously when a difficult airway is encountered. Simulation centers have been demonstrated to provide an effective training platform for this. During any case of difficult airway management, the anesthesiologist should be prepared to rapidly progress to a cricothyroidotomy if this should become necessary and a capnograph should be available to confirm tracheal placement (Figure 10). Careful judgement is also necessary when extubating a patient with a difficult airway. Respiratory depressant drugs like opioids should be used sparingly or avoided altogether and neuromuscular blocking drugs should be fully reversed. Advanced airway equipment should be in close proximity to enable rapid re-intubation if this should become necessary. It is likely that the proactive use of advanced airway equipment will reduce the incidence of airway disasters in the future.

## Figures and Tables

**Table 1:** When to use a VI rather than a DL.

1. When the posterior larynx cannot be visualized even with optimal external laryngeal pressure
2. When laryngeal visualization was poor during past intubation attempts with prior procedures
3. When neck movement during intubation needs to be avoided (trauma, collar *in situ*, cervical spine injury)
4. When muscle relaxants need to be avoided during intubation
5. In patients with temporomandibular joint problems
6. In patients with limited mouth opening when a low profile VL blade has to be used
7. When the pre-op airway exam suggests a poor view at DL

**Note:** VL: Video laryngoscopy; DL: Direct laryngoscopy.
